# CCG implementation of integrated care in the NHS

**DOI:** 10.1186/1472-6963-14-S2-P119

**Published:** 2014-07-07

**Authors:** Jonathan Stokes

**Affiliations:** 1 NIHR Greater Manchester Primary Care Patient Safety Translational Research Centre, University of Manchester, Manchester, UK

## Background

Demographic changes, ageing populations and increasing numbers of patients with multiple long-term conditions (multimorbidity) means health systems must change organisation and delivery to match patient need. Health systems globally are therefore looking to implement ‘integrated care’ as a means to achieve better health system outcomes (health gain, cost-effectiveness, and user satisfaction [1]). The NHS is no exception.

The 2012 Health and Social Care Act, which also created the Clinical Commissioning Groups (CCGs), mandated that these new clinically-led organisations act to support integration of care [2]. However, there is little known about the implementation of integrated care and how CCGs have utilised the flexibility that they have been provided.

This project, therefore, examines a random sample of CCGs and compares the models of integrated care in practice to date.

## Materials and methods

All of the publically available literature from a random sample of 10% (n=21) of the 211 CCGs was examined to determine the models of ‘integrated care’ being implemented.

The model in each CCG was categorised with the aid of an extant health systems framework [1], and models compared across the sample. Results were discussed in terms of innovation displayed by the new CCGs.

## Results

Although the source of information (CCG reports) limited the detail of what could be extracted, there was a clear dominance (n=17/21, 81%) of a single particular model of integrated care present as the primary practice in the NHS. This model can be described as multi-disciplinary case management of high-risk patients, and tends to focus on reducing these patients’ use of acute, secondary care services.

## Conclusions

At the CCG-level, there appears to be a focus on integrating care via ‘service delivery’ interventions, focussed on a small minority of patients determined to be at most risk. The evidence base for this particular intervention is limited at present [3], potentially requiring more justification in terms of health system outcomes.

This clear dominance of a single model also shows limited evidence of innovation, given the potential for flexibility at the CCG-level.

## References

[B1] Atun R Aydin S Chakraborty S Sümer S Aran M Gürol I Nazlioilu S Özgülcü E Aydoian Ü Ayar B Universal health coverage in Turkey: enhancement of equity Lancet 2013 14 65 99 10.1016/S0140-6736(13)61051-X 23810020

[B2] Department of Health Health and Social Care Act 2012 2012

[B3] Ross S Curry N Goodwin N Case Management: What it is and how it can best be implemented King’s Fund; 2011

